# Transcriptome Sequence Analysis of an Ornamental Plant, *Ananas comosus* var. *bracteatus*, Revealed the Potential Unigenes Involved in Terpenoid and Phenylpropanoid Biosynthesis

**DOI:** 10.1371/journal.pone.0119153

**Published:** 2015-03-13

**Authors:** Jun Ma, S. Kanakala, Yehua He, Junli Zhang, Xiaolan Zhong

**Affiliations:** 1 College of Landscape Architecture of Sichuan Agricultural University, Chengdu, Sichuan, China; 2 Institute of Plant Protection, Agricultural Research Organization, the Volcani Center, Bet Dagan, Israel; 3 Horticultural Biotechnology College of South China Agricultural University, Guangzhou, Guangdong, China; Beijing Forestry University, CHINA

## Abstract

**Background:**

*Ananas comosus* var. *bracteatus* (Red Pineapple) is an important ornamental plant for its colorful leaves and decorative red fruits. Because of its complex genome, it is difficult to understand the molecular mechanisms involved in the growth and development. Thus high-throughput transcriptome sequencing of *Ananas comosus* var. *bracteatus* is necessary to generate large quantities of transcript sequences for the purpose of gene discovery and functional genomic studies.

**Results:**

The *Ananas comosus* var. *bracteatus* transcriptome was sequenced by the Illumina paired-end sequencing technology. We obtained a total of 23.5 million high quality sequencing reads, 1,555,808 contigs and 41,052 unigenes. In total 41,052 unigenes of *Ananas comosus* var. *bracteatus*, 23,275 unigenes were annotated in the NCBI non-redundant protein database and 23,134 unigenes were annotated in the Swiss-Port database. Out of these, 17,748 and 8,505 unigenes were assigned to gene ontology categories and clusters of orthologous groups, respectively. Functional annotation against Kyoto Encyclopedia of Genes and Genomes Pathway database identified 5,825 unigenes which were mapped to 117 pathways. The assembly predicted many unigenes that were previously unknown. The annotated unigenes were compared against pineapple, rice, maize, *Arabidopsis*, and sorghum. Unigenes that did not match any of those five sequence datasets are considered to be *Ananas comosus* var. *bracteatus* unique. We predicted unigenes encoding enzymes involved in terpenoid and phenylpropanoid biosynthesis.

**Conclusion:**

The sequence data provide the most comprehensive transcriptomic resource currently available for *Ananas comosus* var. *bracteatus*. To our knowledge; this is the first report on the de novo transcriptome sequencing of the *Ananas comosus* var. *bracteatus*. Unigenes obtained in this study, may help improve future gene expression, genetic and genomics studies in *Ananas comosus* var. *bracteatus*.

## Introduction

Pineapple is a perennial, herbaceous monocot of the family *Bromeliaceae*, sub family *Bromelioideae*, in the order *Poales*. According to the recent classification, Coppens d’Eeckenbrugge and Leal, proposed one genus, *Ananas*, with two species *A*. *comosus* (L.) Merr. (diploid, 2n = 2x = 50) and *A*. *macrodontes* Morren (tetraploid, 2n = 4x = 100). *A*. *comosus* is subdivided into five botanical varieties: var. *comosus*, var. *ananassoides*, var. *erectifolius*, var. *parguazensis* and var. *bracteatus* [1].

Pineapple is native to South America, cultivated commercially in the tropics and subtropics parts of the world. It is considerable economic and horticultural crop of the world harvested over an area of 0.9 million ha with a production of about 23.3 million tonnes in 2012 (http://faostat.fao.org). Pineapple represents the most valuable food sources consumed globally as fresh/canned juice, mainly in vegetable diets. Traditionally, pineapple crop was grown for its stem and leaves, which were a source of high quality silk fiber, widely used in paper and clothes industry [2,3]. It is also source of bromelain, a proteolytic enzyme complex, used in the meat industry, but due to its health benefits, it is now commercially used in pharmaceutical industry, since they contain substances and vitamins that are beneficial for human health [4]. Among the five varieties of *A*. *comosus*, *A*. *comosus* var. *bracteatus* is cultivated as ornamental plants for its colorful leaves and decorative red fruit.

Several plants have the capacity to synthesize a huge reach of organic compounds that are traditionally classified as primary and secondary metabolites. Primary metabolites are compounds, which play essential roles in photosynthesis, respiration, normal development, evolution and reproduction. In contrast, secondary metabolites often play significant functions in plant defense. To date, a large number of secondary metabolites were synthesized from *Ananas* leaves and fruit infusions [5–7]. These include flavonoids, terpenoids and alkaloids, which contribute color to the leaves and fruit and also commonly used as new natural drugs, antibiotics, insecticides and weed killers [8,9]. A recent study of phytochemical analysis for *A*. *comosus* peel confirmed the presence of phenols, flavonoid and alkaloid [10]. The abundance of secondary metabolites makes *A*. *comosus* var. *bracteatus* a good model for investigating the flavonoid and terpenoid biosynthesis in plants and the related genes and pathways. Therefore an increasing interest has been excited to experience more about the secondary metabolism products of *A*. *comosus* var. *bracteatus*, could help us amend the employment of these natural products for human uses.

In recent years, researchers have concentrated on studying the genetic diversity of the genus *Ananas*. Expressed sequence tags (ESTs) sequencing has traditionally been the core technology used for discovery of reference gene transcripts. For example, ESTs were generated from cDNA libraries of *A*. *comosus* var. *comosus* roots, fruit and aerial tissues [11], green mature fruits [12] and nematode infected gall [13]. However, it has some inherent limitations, such as time consuming, cloning, cDNA library construction, and many Sanger sequencing runs. Later, gene expression microarray results have produced much important information about how the transcriptome is deployed in different cell types [14] and tissues [15]. In 2012, first time microarray based gene expression study undertaken in pineapple [16]. This study identified a number of genes, processes and pathways with putative involvement in the pineapple fruit ripening process [16]. Following this sequencing, Ong *et al*. generated 4.7 million paired-end high quality reads of ripe yellow *A*. *comosus* var. *comosus* fruit using Illumina technology [17]. The assembly produced 28,728 unique transcripts with average length of approximately 200 bp. A total 16,932 unique transcripts were identified against non-redundant NCBI database. Of these 15,507 unique transcripts were assigned to gene ontology terms and 13,598 unique transcripts were mapped to 126 pathways in the genomes pathway database (Kyoto Encyclopedia of Genes and Genomes; http://www.genome.jp/kegg/). To date, however, the *A*. *comosus* var. *bracteatus* genome has not been fully sequenced to understand the underlying functional mechanisms and its encoded genes. As October 2014, only 110 nucleotide sequences, 0 ESTs, 3 protein and 0 genes from *A*. *comosus* var. *bracteatus* had been deposited in the NCBI’s GenBank database.

In the present study, a *de novo* transcriptome sequencing for *A*. *comosus* var. *bracteatus* using the Illumina sequencing was initiated. Leaf, root and stem samples of *A*. *comosus* var. *bracteatus* were sequenced and a total of 23,584,613 (23.5 million) reads and 41,052 unigenes were identified. However, short read length limits *de novo* contig assembly efficiency. Discussions on sequencing bias of high-throughput technologies have taken place in several publications [18–21]. To our knowledge, this is the first transcriptome characterization of *A*. *comosus* var. *bracteatus*. In addition, we compared transcriptome sequence similarity with pineapple (*Ananas* sp.), maize (*Zea mays*), sorghum (*Sorghum bicolor*), rice (*Oryza sativa*), *Arabidopsis* (*Arabidopsis thaliana*). This information provides genes and gene network involved in the significant biological and economical characters of *A*. *comosus* var. *bracteatus*.

## Results and Discussion

### Sequencing and *de novo* transcriptome assembly

**Fig 1:**
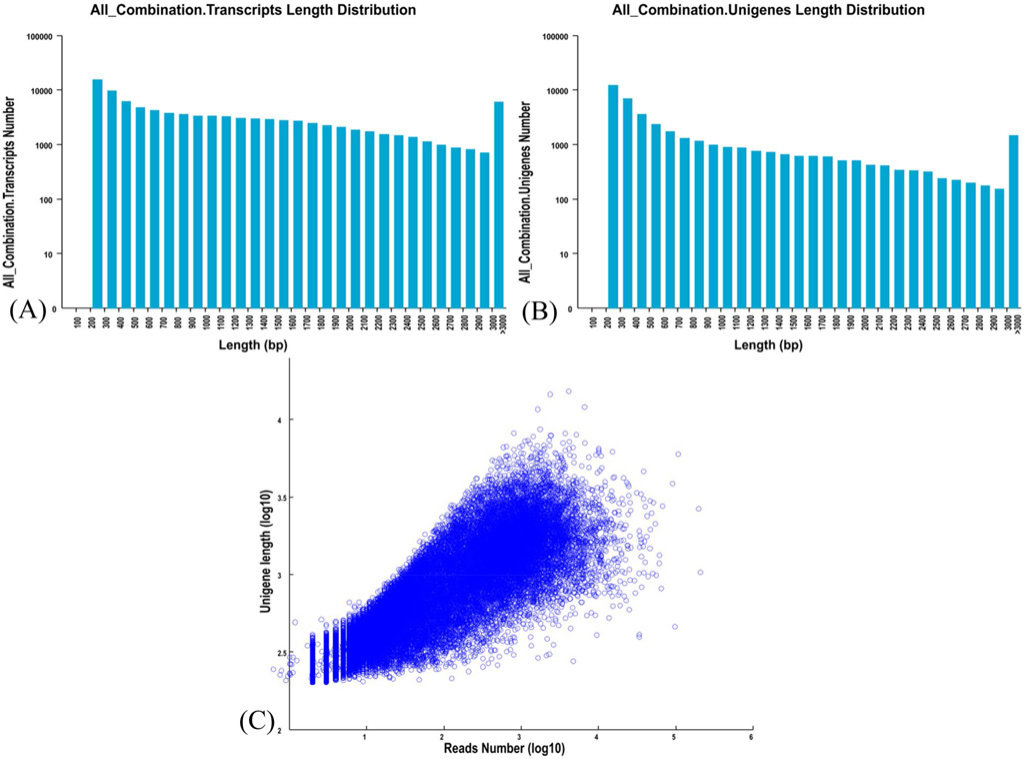
Overview of the *A*. *comosus* var. *bracteatus* transcriptome sequencing and assembly. (A) Length distribution of *A*. *comosus* var. *bracteatus* transcripts. (B) Size distribution of *A*. *comosus* var. *bracteatus* unigenes. (C) Log-log plot showing the dependence of unigene lengths on the number of reads assembled into each unigene.

The cDNA library for *A*. *comosus* var. *bracteatus* was prepared and sequenced using the Illumina Genome analyzer. In order to accurately analyze the data, we filter the raw data to ensure the quality of each reads the value of less than 20 nucleotides does not exceed 20%, N content of not more than 5% removal of ribosomal RNA. After data filtering, 23,584,613 (23.5 million) reads with 98.64% Q20 bases were selected as high quality reads for further analysis. After the removal of adaptor sequences and exclusion of contaminated or short reads, 23.5 million high quality read sequences were assembled into 1,134,553,18 contigs using SOAPdenovo [22]. Using the Trinity *de novo* assembly program, short-reads were assembled into 1,186,576,93 transcripts with a mean length of 1224.83 bp (Fig. 1A). The transcripts were subjected to cluster and assembly analyses. A total 41,052 unigenes were obtained, with an average length of 837.62 bp, among which 10,923 genes (26.61%) were greater than 1 kb (Fig. 1B). These results demonstrated the effectiveness of Illumina sequencing technology in rapidly capturing a large portion of the transcriptome.

The length distributions of contigs, transcripts and unigenes are shown in Table 1. The N50 values of transcripts and unigenes were 1869 and 1520 bp, respectively. As expected for a randomly fragmented transcriptome, there was a positive relationship between the length of a given unigene and the number of reads (Fig. 1C). To facilitate the access and use of *A*. *comosus* var. *bracteatus*, transcriptome sequencing data have been deposited in the NCBI Sequence Read Archive (SRA) database with accession number SRX681749.

**Table 1:** Summary of Illumina transcriptome assembly for *A*. *comosus* var. *bracteatus*.

Length range	Contigs	Transcripts	Unigenes
**200–300**	1,521,010 (97.76%)	15,445 (15.94%)	12,186 (29.68%)
**300–500**	13,648 (0.88%)	15,849 (16.36%)	10,466 (25.49%)
**500–1000**	9,780 (0.63%)	19,596 (20.23%)	7,477 (18.21%)
**1000–2000**	7,504 (0.48%)	27,260 (28.51%)	6,696 (16.31%)
**2000+**	3,866 (0.25%)	18,367 (18.96%)	4,227 (10.30%)
**Total number**	1,555,808	96,877	41,052
**Total length**	113,455,318	118,657,693	34,386,147
**N50 length**	94	1,869	1,520
**Mean length**	72.92	1224.83	837.62

### Sequence annotation

In order to make an functional annotation and classification for the putative identities of the assembly, all the unigenes were annotated by aligning with deposited sequences in diverse protein databases including the National center for Biotechnology (NCBI), nonredundant protein (Nr) database, NCBI non-redundant nucleotide sequence (Nt) database, UniProt/Swiss-Prot, Kyoto Encyclopedia of Genes and Genomes (KEGG), Cluster of Orthologous Groups of proteins (COG) and UniProt/TrEMBL. The best one was selected from the matches with an E-value of less than 10^-5^. Only 24,749 (60.2%) unigenes were annotated with a threshold of 10^-5^ by performing a BLASTx using above diverse protein databases (Nr, Nt, Swiss-Port, COG, KEGG and TrEMBL). According to the BLASTx results, 23,275 (56.6%) unigenes have homologues proteins in the Nr protein database, and 19, 817 (48.2%) had significant matches in Nt database and while 17,579 (42.8%) unigenes had similarity to proteins in the Swiss-Port database are listed in S1 Table. The overall functional annotation is listed in Table 2. Out of 41,052 unigenes, only 24,749 (60.2%) unigenes matched to known genes while 16,303 (39.7%) unigenes were unmapped in those databases, which could be attributable to the short reads generated by the sequencing technology, most of which probably lack the conserved functional domains or insufficient sequences of pineapple in the public databases.

**Table 2:** Functional annotation of the *A*. *comosus* var. *bracteatus* transcriptome.

Annotated databases	All sequence	> = 300nt	> = 100nt
**COG**	8,505	7,522	4,837
**GO**	17,748	15,102	8,482
**KEGG**	5,825	4,930	2,840
**TrEMBL**	17,579	15,034	8,252
**Swiss-port**	23,134	19,619	10,444
**NR**	23,275	19,569	10,343
**NT**	19,817	16,881	9,671
**All**	24,749	20,403	10,516

**Fig 2:**
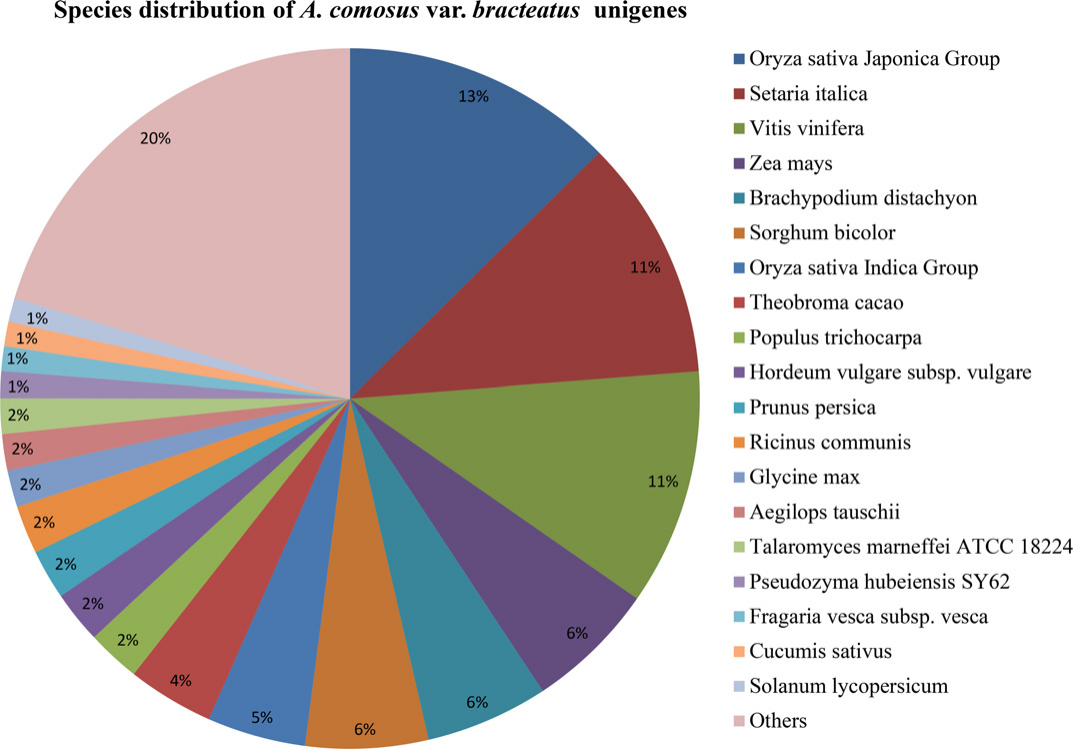
Species distribution of *A*. *comosus* var. *bracteatus* from the velvet *de novo* assembly.

Based on the above similarity search, we conducted species distribution of pineapple unigenes based on the BLASTx analysis, it showed that the unigenes hit a range of plant species. Among the various plants that have protein sequences in GenBank, the *A*. *comosus* var. *bracteatus* unigenes had the highest number of homologous to *Oryza sativa* Japonica group (13%), *Setaria italica* (11%) and *Vitis vinifera* (11%). This was followed by *Zea mays* (6%), *Brachypodium distachyon* (6%), *Sorghum bicolor* (6%), *Oryza sativa* Indica group (5%), *Theobroma cacao* (4%). While less than 4% homologues to other plants and others (20%) were summarized in the Fig. 2. Interestingly, *A*. *comosus* var. *comosus* fruit transcriptome also had the highest similarities (26.2%) with *O*. *sativa* [17]. The high similarities of the *A*. *comosus* var. *comosus* [17] and *A*. *comosus* var. *bracteatus* (this study) unigenes to the *O*. *sativa* genes may provide for the possibility of using the rice EST’s as a reference for future assembly using next generation sequencing.

**Fig 3:**
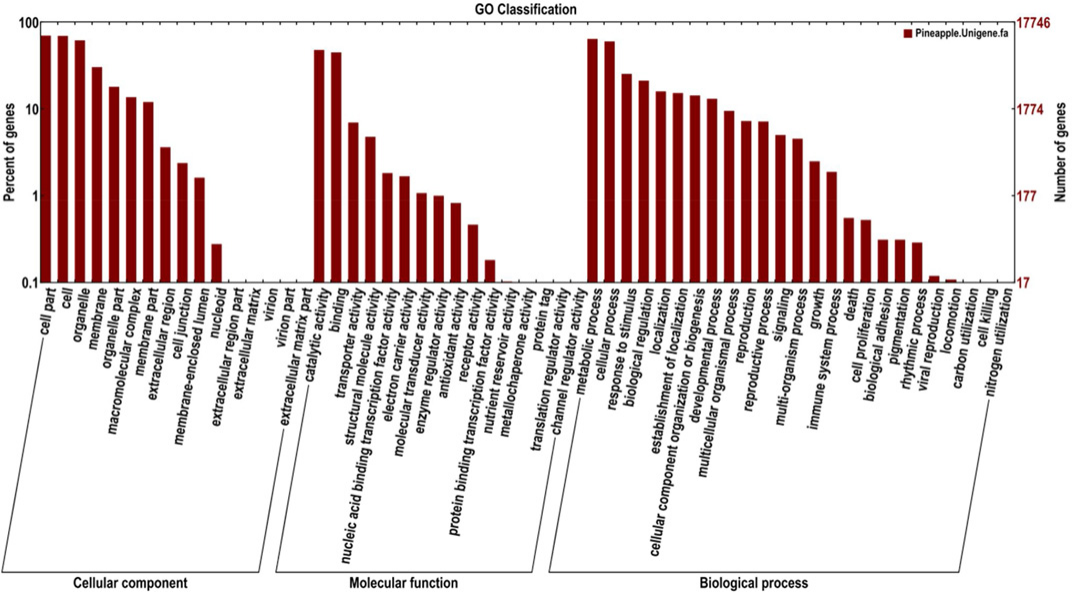
Functional annotation of assembled sequences based on gene ontology (GO) categorization. GO analysis was performed at the level two for three main categories (cellular component, molecular function and biological process).

In addition, functions of the assembled transcripts were classified using Gene Ontology (GO). A total of 17,746 unigenes were divided into three ontologies: cellular component, molecular function and biological processes. Of these, majority of the GO terms were assigned to biological processes (47%), followed by cellular component (27%) and molecular function (26%) (Fig. 3). The biological function category mainly comprised proteins involved in metabolic, cellular processes and response to stimuli, biological regulation and localization and biogenesis are most represented functions. Of these, genes involved in the metabolic and cellular processes were highly represented. In cellular components, the major classifications of these genes products were cell, cell part, organelle and membrane. For molecular functions, the genes involved in catalytic and binding activities were both highly represented. In comparison to pineapple fruit GO annotation [17], more number of the genes were expressed in all the three ontologies (cellular component, molecular function, biological function). GO annotation provided a general gene expression profile picture for *A*. *comosus* var. *bracteatus*, which showed that the sequenced genes were responsible for fundamental, biological regulation and metabolism.

**Fig 4:**
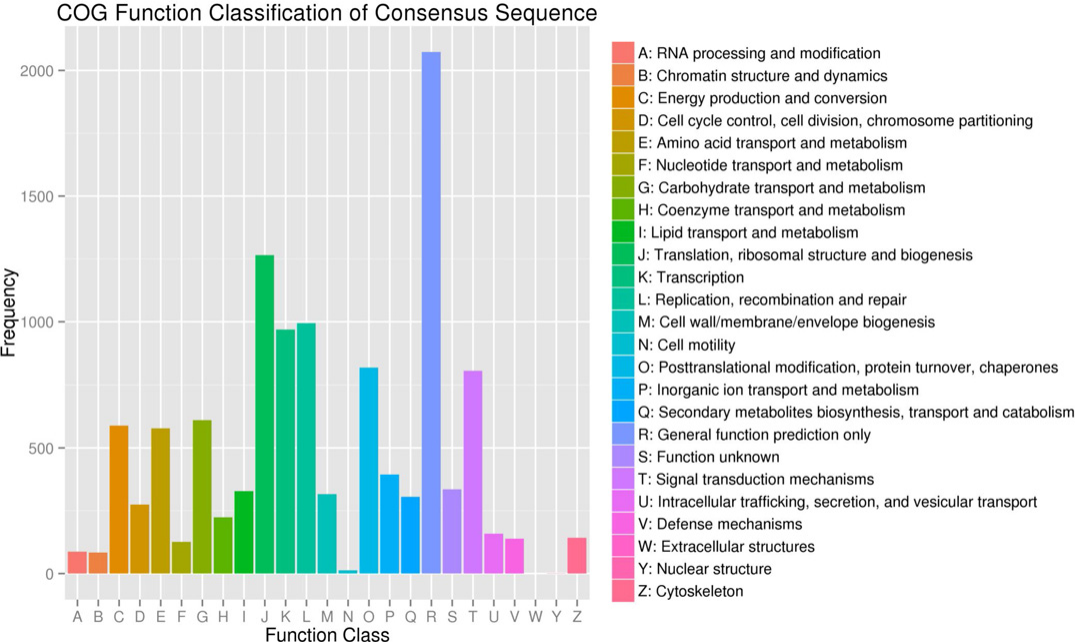
Clusters of orthologous groups (COG) classification. In total, 8,505 of the 41,052 sequences with Nr hits were grouped into 25 COG classifications.

Based on the Nr annotation, all unigenes were subjected to a search against the COG database for functional prediction and classification. In total, 8,505 unigenes with hits in Nr database could be assigned to COG classification and divided in to 25 specific categories, listed in Fig. 4. The cluster for general function prediction (17.82%) represented the largest group, followed by translation, ribosomal structure and biogenesis (10.87%), replication, recombination and repair (8.54%) and transcription (8.33%), posttranslational modification, protein turnover, chaperones (7.05%) and signal transduction mechanisms (6.94%). Approximately 5% unigenes hit with energy production and conversion, carbohydrate transport and metabolism and amino acid transport and metabolism. However, unigenes involved in secondary metabolite biosynthesis were found in the categories of energy production and conversion and secondary metabolites biosynthesis, transport and catabolism respectively.

The KEGG database can be used to categorize gene functions with an emphasis on biochemical pathways. To better understand biological pathways in *A*. *comosus* var. *bracteatus*, a BLASTx search against the KEGG protein database was made on the assembled unigenes. This process predicted a total of 5,825 (14.19%) were grouped into 117 pathways. Summary of the sequences involved in these pathways were included in S2 Table. The predicted pathways represented the majority of ribosome (Ko03010, 629 unigenes, 9.63%), oxidative phosphorylation (Ko00190, 274 unigenes, 4.20%), protein processing in endoplasmic reticulum (Ko04141, 199 unigenes, 3.05%) and glycolysis/gluconeogenesis (Ko00010, 194 unigenes, 2.97%). A total of 131 (2.01%) transcripts containing the C3 and CAM pathways were found to be potentially related to carbon fixation in *A*. *comosus* var. *bracteatus*. Other important pathways with highest transcripts mapping include RNA transport (Ko03013, 172 unigenes, 2.63%), Spliceosome (Ko03040, 171 unigenes, 2.62%), purine metabolism (Ko00230, 159 unigenes, 2.43%) and plant hormone signal transduction (Ko04075, 150 unigenes, 2.30%). Besides all these pathways, phenylpropanoid biosynthesis (Ko00940, 92 unigenes, 1.41%), terpeniod backbone biosynthesis (ko00900, 47 unigenes, 0.72%) and flavonoid biosynthesis (Ko00941, 25 unigenes, 0.38%) were most enriched pathways clearly suggested that the plant possess different secondary metabolites with different metabolic functions in plants. In addition, genes involved in Porphyrin and chlorophyll metabolism pathways (Ko00860, 45 unigenes, 0.69%) represented the biological processes of energy capture and utilization in *A*. *comosus* var. *bracteatus*. Moreover, few genes were also involved in riboflavin metabolism, vitamin B6 metabolism, biotin metabolism, lipoic metabolism and thiamine metabolism pathways in the unigene collection.

### Comparative analysis with pineapple, rice, maize, sorghum and *Arabidopsis*

**Fig 5:**
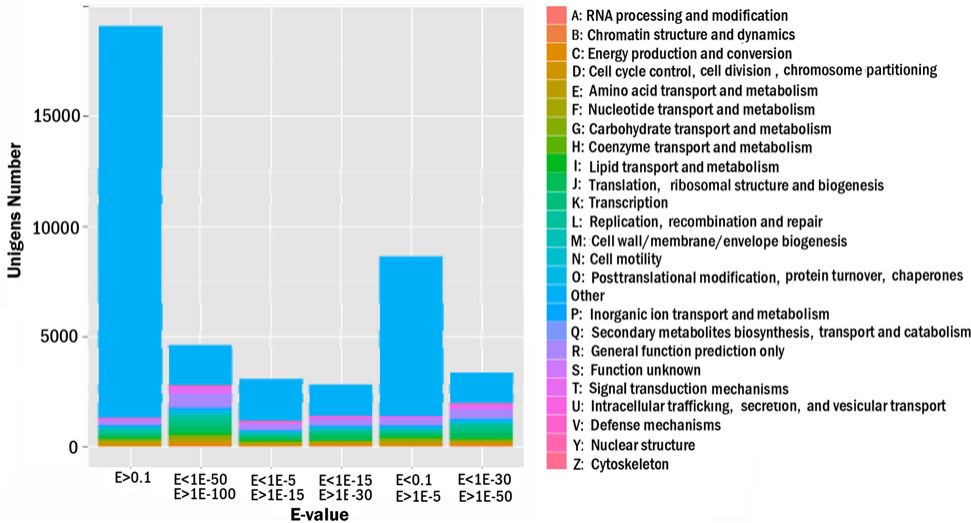
Distribution of Conservation between *A*. *comosus* var. *bracteatus* unigenes and pineapple EST sequences based on tBLASTX scores.

In our transcriptome, we examined *A*. *comosus* var. *bracteatus* unigenes with five species namely pineapple, maize, sorghum, rice and *Arabidopsis* available genome data. To study the distribution of conversation between *A*. *comosus* var. *bracteatus* unigenes and pineapple EST sequences obtained from NCBI database (until October 2014) based on tBLASTx method. For this analysis, the E-values of tBLASTx similarity searches were used an estimate of sequence conservation. The detailed list of tBLASTx scores are listed in the S3 Table. Based on the above distribution of conservation search, we simultaneously examined more closely the putative functional role between *A*. *comosus* var. *bracteatus* unigenes and available pineapple EST sequences (Fig. 5).

**Fig 6:**
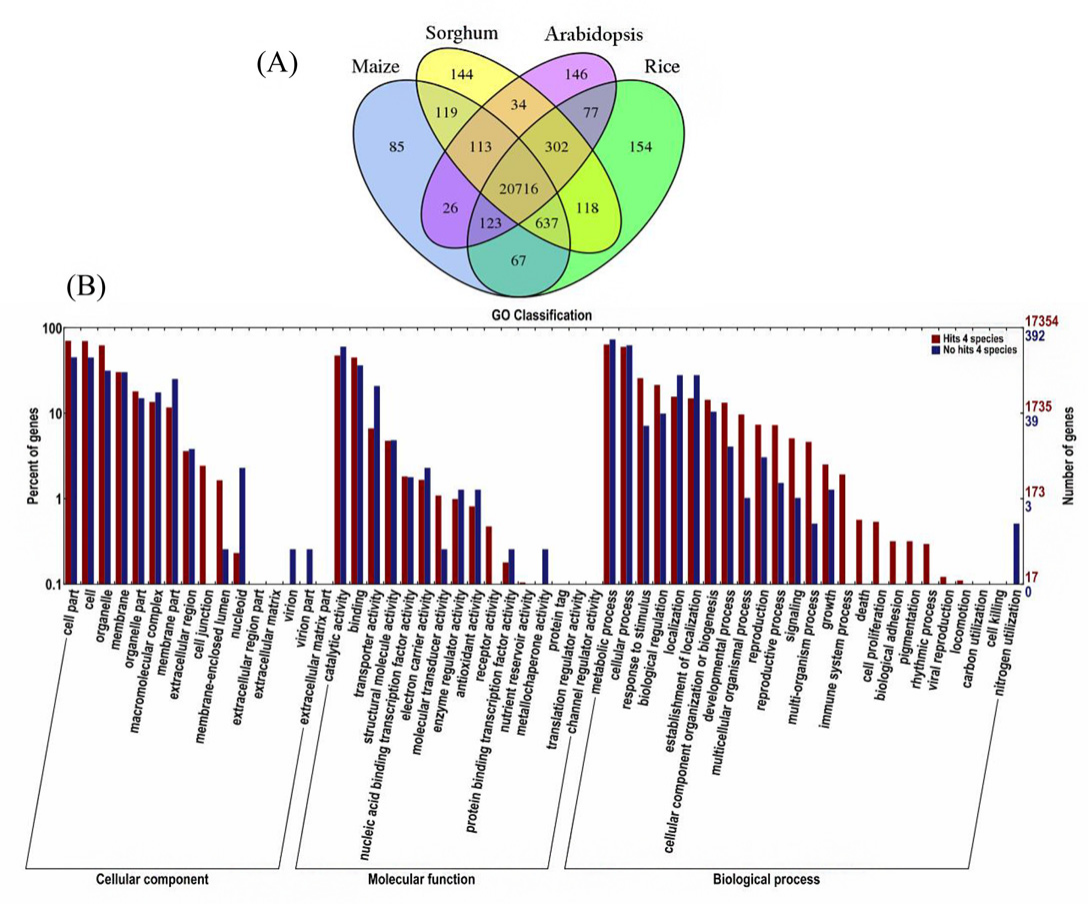
*A*. *comosus* var. *bracteatus* unigene similarity comparison with rice, maize, sorghum and *Arabidopsis* and functional classification by GO analysis. (A) Similarity search of *A*. *comosus* var. *bracteatus* sequence against rice, maize, sorghum and *Arabidopsis*. (B) Functional classification of *A*. *comosus* var. *bracteatus* unigenes with and without homologs with rice, maize, sorghum and *Arabidopsis*.

To study the sequence conservation of *A*. *comosus* var. *bracteatus* unigenes in other plant species, a BLASTX search was performed against the four available proteomes of completely sequenced plant genomes. The largest number of *A*. *comosus* var. *bracteatus* unigenes exhibited 22,194 significant similarity hits to rice transcript, 21,886 unigenes had similarity hits to maize, 22,183 unigenes had similarity hits to sorghum and 21,537 unigenes had similarity hits to *Arabidopsis* (Fig. 6A). Based on the above similarity search, we then conducted GO analysis to compare the functional classification between *A*. *comosus* var. *bracteatus* and the above five species (Fig. 6B). The detailed results were listed in S4 Table. A total of 20,716 shared homologues among these four species. Of these, genes involved in metabolic and cellular processes are highly represented in biological processes. Under the molecular function ontology, proteins involved in binding and catalytic activity was dominant while in cellular component ontology, proteins for cell part, cell and organelle development were generally encoded by *A*. *comosus* var. *bracteatus* unigenes. Therefore, we conclude that the differences in the expression profile and functional allocation of the *A*. *comosus* var. *bracteatus* unigenes with sequence similarity hits to four species concurrently contribute to the divergence of *A*. *comosus* var. *bracteatus* from other crops.

### Terpeniod backbone biosynthesis

**Fig 7:**
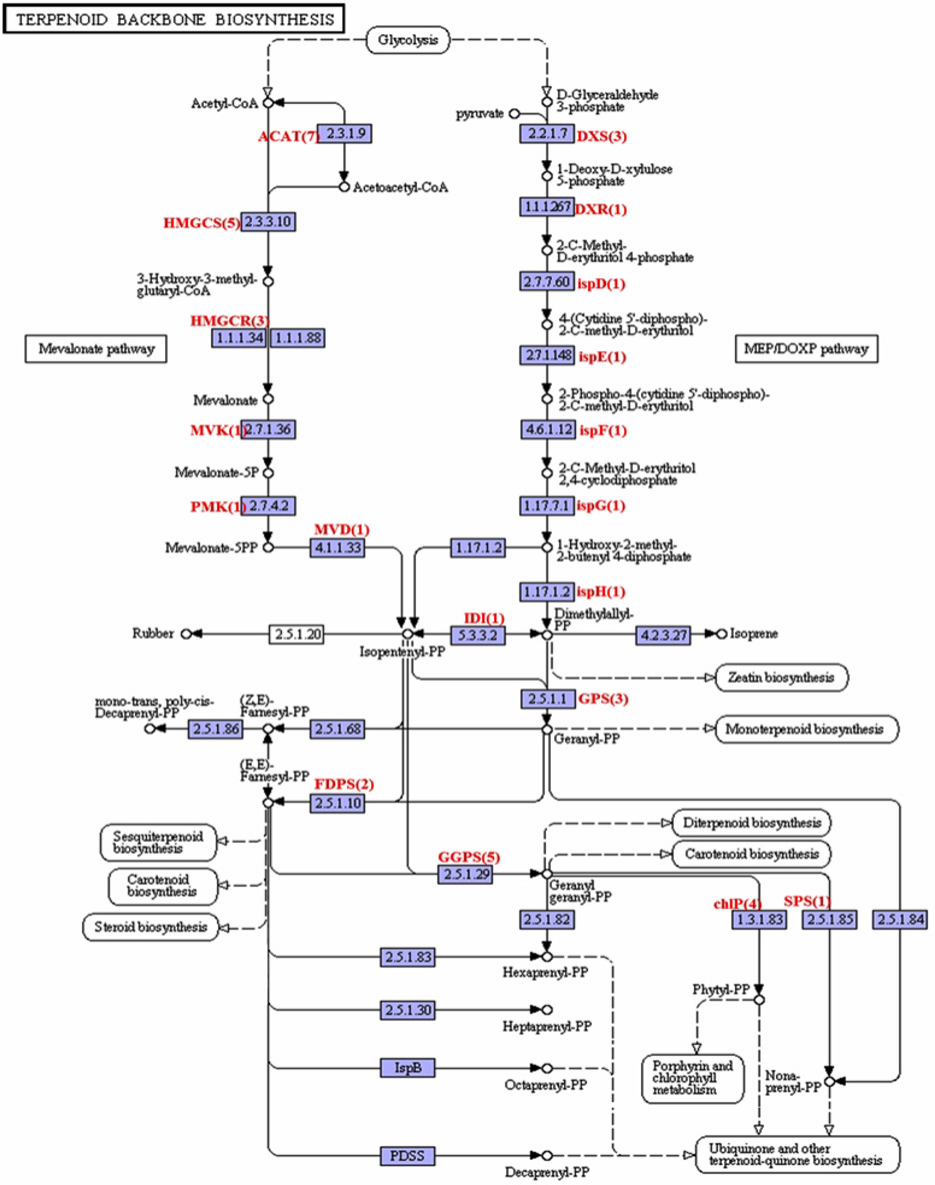
The Terpenoid backbone biosynthesis. The *A*. *comosus* var. *bracteatus* transcripts encoding enzymes (highlighted) involved in the pathway was identified by BLASTx (E-value of less than 10^-5^). Numbers in bracket are the total number of unigenes encode for the enzyme.

Terpenoids, are the largest group of natural products, extensively used for their aromatic qualities and for the treatment of human diseases [23]. All terpenoids are synthesized through two independent pathways: the cytosolic mevalonic acid (MVA) pathway and the plastidialmethyerythriol phosphate (MEP) pathway (Fig. 7). In our transcriptome, we identified 43 unigenes, which encode all enzymes involved in the terpeniod backbone biosynthesis.

The mevalonate pathway starts with formation of two molecules of acetyl CoA to produce acetoacety CoA condensation by acetyl-CoA C-acetyltransferase (ACAT, EC 2.3.1.9, 7 unigenes). ACAT is then condensed with a third molecuale of acetyl CoA in an aldol-like reaction catalyzed by hydroxymethylglutaryl-CoA synthase (HMGCS, EC 2.3.3.10, 5 unigenes). Hydrolyzed HMGCS to give Mevalonate byhydroxymethylglutaryl-CoA reductase (NADPH) (HMGCR, EC1.1.1.34, 3 unigenes). The mevalonate kinase (MVK) with the EC number of 2.7.1.36 was encoded by 1 unigene react to convert mevalonate to mevalonate-5-pyrophosphate. A final phosphorylation at the tertiary hydroxyl group catalyzed by phosphomevalonate kinase (PMK, EC 2.7.4.2, 1 unigene) and mevalonate-5-diphosphate decarboxylase (MVD, EC 4.1.1.33, 1 unigene) which results in the production of 3-isopentenyl pyrophosphate.

Another important pathway, in addition to isopentenyl pyrophosphate biosynthesis is MEP/DOXP pathway. In the first step, the pyruvate is converted into 1-deoxy- D-xylulose-5-phosphate catalyzed by 1-deoxy- D-xylulose-5-phosphate synthase (DXS, EC 2.2.1.7, 3 unigenes). Next, 1-deoxy- D-xylulose-5-phosphate is catalyzed by deoxyxylulose-5-phosphate reductoisomerase (DXR, EC 1.1.1.267, 1 unigene) to produce methyl- D-erythritol 4-phosphate (MEP). MEP is converted into isopentenyl diphosphate through a number of enzymatic reactions. The enzymes involved were found to be encoded by the unigenes are 2-C-methyl-D-erythritol 4-phosphate cytidylyltransferase (ispD, EC 2.7.7.60, 1 unigene), 4-(Cytidine 5’-diphospho)-2-C-methyl-D-erythritol kinase (ispE, EC 2.7.1.148, 1 unigene), 2-C- methyl-D-erythritol 2,4-cyclodiphosphate synthase (ispF, EC 4.6.1.12, 1 unigene), 1-hydroxy-2-methyl-2-(E)-butenyl 4-diphosphate synthase (ispG, EC 1.17.7.1, 1 unigene), 4-hydroxy-3-methylbut-2-enyl diphosphate reductase (ispH, EC 1.7.1.2, 1 unigene) and isopentenyl-diphophate Delta-isomerase (IDI, EC5.3.3.2, 1 unigene).

Next, conversion of isopentenyl diphosphate or dimethylallyl diphosphate to geranyl diphosphate (geranyl-PP) was carried out by dimethylallyltransferase (geranyl geranyl diphosphate synthase (GPS), EC 2.5.1.1, 3 unigenes) for the synthesis of monoterpens. The enzymes, farnesyldiphospahate synthase (FDPS, EC 2.5.1.10, 2 unigenes) and geranyl geranyl diphosphate synthase (GGPS, EC 2.5.1.29, 5 unigenes), are involved in the synthesis of farnesyl diphospahte (farnesyl-PP) and geranyl geranyl diphosphate (geranyl geranyl-PP) respectively. The enzymes which are responsible for synthesis of sesquiterpenes, caretenoid, steroid, porphyrin and chlorophyll metabolism, ubiquinone and other terpenoid-quinone from farnesyl-PP and geranyl geranyl-PP were identified in *A*. *comosus* var. *bracteatus* sequence assembly (Fig. 7). Transcriptome sequencing studies of Ong *et al*. [17] and our dataset results indicate the clear picture of terpenoid backbone synthesis in the genus *Ananas*. The EC number and number of unigenes encoding the enzymes involved in the terpenoid backbone biosynthesis are listed in the S5 Table.

### Phenylpropanoid biosynthesis

The phenylpropanoid pathway serves as a rich source of metabolites in plants, being required for biosynthesis of lignin, flavonoids, coumarins and hydroxycinnamic acids [24,25]. In our annotated *A*. *comosus* var. *bracteatus* transcriptome datasets, we identified multiple unigenes encoding enzymes involved in known lignin and flavonoid pathways. Because of pharmacological effects of *A*. *comosus* var. *bracteatus* lignin and flavonoids, we focused on the related genes in this study.

**Fig 8:**
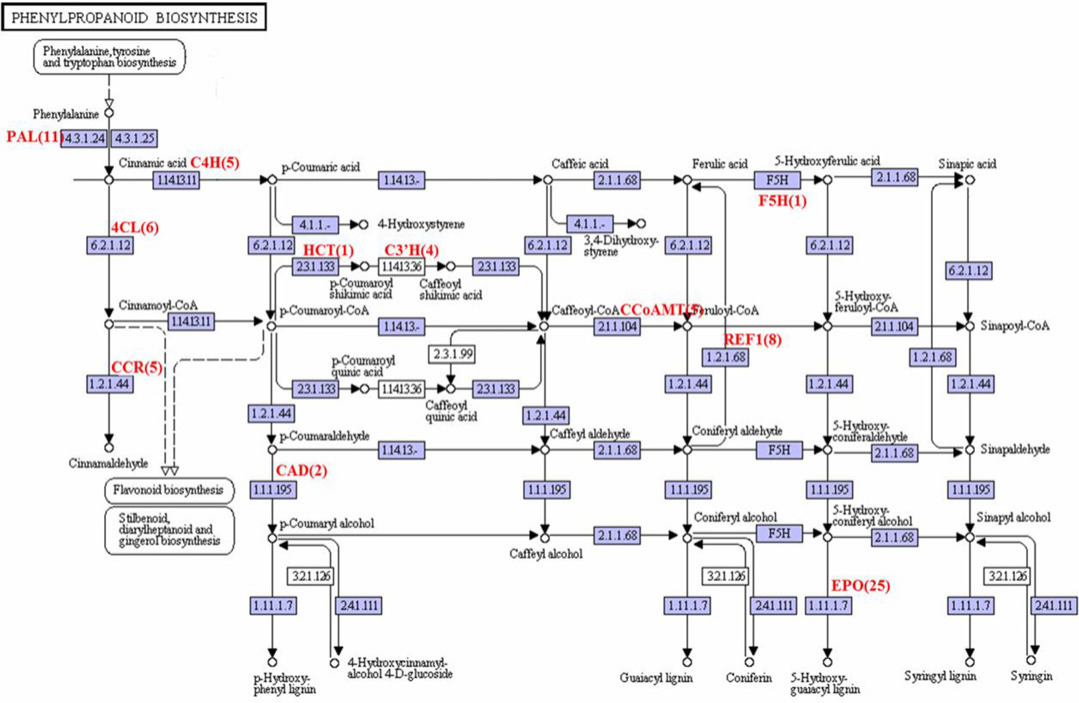
The Phenylpropanoid biosynthesis. The A. comosus var. bracteatus transcripts encoding enzymes (highlighted) involved in the pathway was identified by BLASTx (E-value of less than 10–5). Numbers in bracket are the total number of unigenes encode for the enzyme.

As is well known, lignin plays a crucial role in plant development and prevents pathogen invasion [26]. Aside from the structural function, lignin derivatives have been shown to have several bioactive functions. Recently, Ito et al. reported that lignin derivatives suppress the apoptosis of neural cells caused by oxidative stress [27]. The initial step in its biosynthesis involves the deamination of phenylalanine to cinnamic acid by phenylalanine ammonia lyase (PAL, EC 4.3.1.24, 11 unigenes) (Fig. 8). Cinnamic is converted into ferulic and sinapic acids by trans-cinnamate 4-monooxygenase (C4H, EC 1.14.13.11, 5 unigenes), coniferyl-aldehyde dehydrogenase (REF1, EC 1.2.1.68, 8 unigenes) and ferulate 5-hydroxlase (F5H, EC 1.14.-.-, 1 unigene). These acids are then ligated to CoA, leading to the synthesis of CoA acid derivatives (caffeoyl-CoA, Feruloyl-CoA, 5-hydroxy-feruloyl-CoA and sinapoyl-CoA) by 4-coumarate-CoA ligase (4CL, EC6.2.1.12; 6 unigenes). Other intermediate enzyme, caffeoyl-CoA O-methyltransferase (CCoAMT, EC 2.1.1.104, 5 unigenes) convert caffeoyl-CoA to Feruloyl-CoA and 5-hydroxy-feruloyl-CoA to sinapoyl-CoA. Next, cinnamoyl-CoA reductase (CCR, EC1.2.1.44, 5 unigenes) converts acids into caffeoyl, Feruloyl, 5-hydroxy-feruloyl and sinapoyl aldehydes. All these aldehydes synthesized above then reduced to alcoholic compound bycinnamyl-alcohol dehydrogenase (CAD, EC: 1.1.1.195; 2 unigenes). In the final step, aldehydes are polymerized into p-Hydroxyphenyl lignin, Guaiacyl lignin, 5-Hydroxyguaiacyl lignin and syringyl lignin by peroxidase (EPO, EC 1.11.1.7, 25 unigenes). The EC number and number of unigenes encoding the enzymes involved in the phenylpropanoid biosynthesis are listed in the S5 Table.

**Fig 9:**
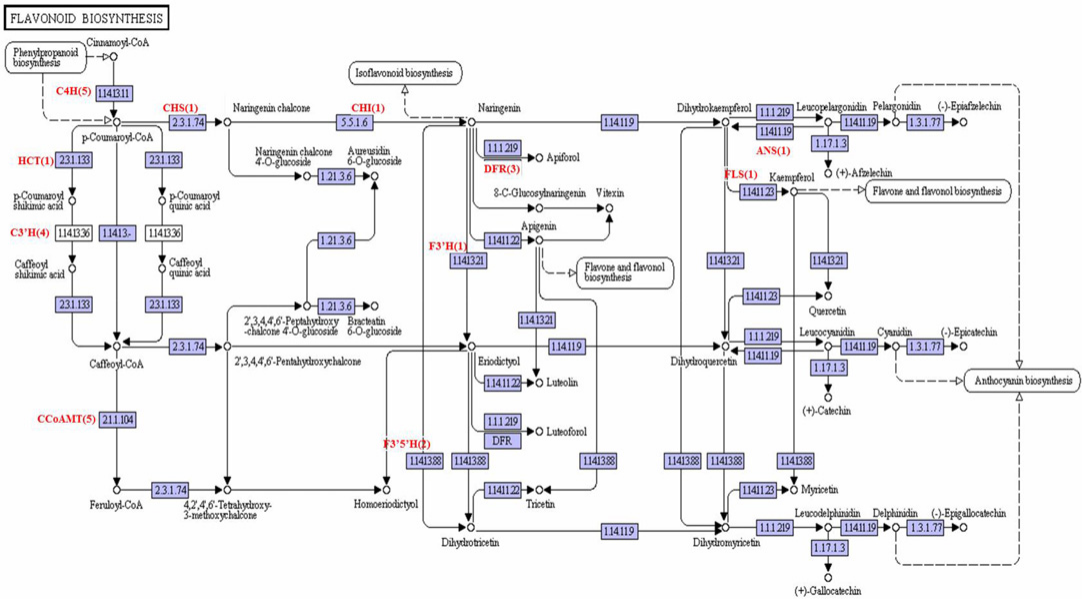
The Flavonoid biosynthesis. The *A*. *comosus* var. *bracteatus* transcripts encoding enzymes (highlighted) involved in the pathway was identified by BLASTx (E-value of less than 10^-5^). Numbers in bracket are the total number of unigenes encode for the enzyme.

Unigenes involved in the flavonoid biosynthesis were also present in *A*. *comosus* var. *bracteatus* transcriptome dataset. Flavoniods, are a class of plant secondary metabolites, play a vital biological and pharmacological activities in *in vitro* studies [28]. The flavonoid biosynthetic pathways have already have been reported in snapdragon (*Antirrhinum majus*), petunia (*Petunia hybrida*) *A*. *thaliana*, *Z*. *mays* and *V*. *vinifera* [29,30]. Flavonoids are synthesized via the phenylpropanoid pathway, where the phenylalanine is used to produce 4-coumaryol-CoA, and this then combined with malonyl-CoA to produce chalcones which are backbones of flavonoids (Fig. 9). The first step involves the activity of four enzymes, phenylalanine ammonia lyase (PAL, EC 4.3.1.24, 11 unigenes), cinnamate 4-hydroxylase (C4H, EC 1.14.13.11, 5 unigenes), 4- coumarate CoA ligase (4CL, EC 6.2.1.12, 6 unigenes), and chalcone synthase (CHS, EC 2.3.1.74, 1 unigene), which converts phenylalanine to chalcone. Chalcone isomerase (CHI, EC 5.5.1.6, 1 unigene) then catalyzes the isomerisation of chalcones into naringenin. Subsequently, Naringenin is converted into flavonones, eriodictyol and dihydrotricetin by two enzymes namely, flavonoid 3’- hydroxylase (F3’H, EC 1.14.13.21, 1 unigene) and flavonoid 3’, 5’-hydroxylase (F3’5’H, EC 1.14.13.88, 2 unigenes) respectively. Flavanone 3-hydroxylase (F3H, EC 1.14.11.9) which converts flavanones to dihydroflavonols was not detected in annotated unigenes. Dihydroflavonols can then lead to production of flavonols and flavan—3, 4-diols (leucoanthocyanidin), reactions being catalysed by flavonol synthase (FLS, EC 1.14.11.23, 1unigene) and by dihydroflavonol 4-reductase (DFR, EC 1.1.1.219, 2 unigenes) respectively. The leucoanthocyanidins can either be converted to anthocyanidins by leucoanthocyanidin dioxygenase (ANS, EC 1.14.11.19, 1 unigene). We also failed to find any Anthocyanidin reductase (LAR, EC 1.17.1.3) which is responsible for production of catechins, however, we did identify one leucoanthocyanidin dioxygenase (ANS), suggesting that the genes encoding other anthocyanidine-related products may exist but have not yet been identified in the *A*. *comosus* var. *bracteatus*. The Anthocyanin and flavones formed here are important pigments for the beautiful red color of the leaf and fruit of *A*. *comosus* var. *bracteatus*. Unigenes of *A*. *comosus* var. *bracteatus* encoding twelve enzymes in the flavonone biosynthesis pathway are listed in S5 Table.

### Conclusions

In recent years transcriptome sequencing has become an effective tool to discover the unigenes at a given moment in time. The present work presents the first de novo transcriptome sequencing analysis using RNA-seq technology to profile the *A*. *comosus* var. *bracteatus* transcriptome on the Illumina platform. A total, 23,584,613 high-quality reads were obtained from the platform, and 41,052 unigenes were identified by *de novo* assembly. Among them, 24,749 (60.2%) unigenes were annotated indicating their relatively conserved functions. Functions of the unigenes were classified by COG, GO and found potentially involved in biological, cellular and molecular processes. In addition, *A*. *comosus* var. *bracteatus* genes related to terpenoid and phenylpropanoid pathways were characterized and their sequences compared to the sequence databases of pineapple, maize, sorghum, rice and *Arabidopsis*. To our knowledge, this is the first report on the characterization of the transcriptome of *A*. *comosus* var. *bracteatus* and assembly of the high quality reads was conducted without a reference genome. This work will provide a sequence source and facilitate the studies in molecular mechanism of terpenoid, lignin and flavonoid biosynthesis in *A*. *comosus* var. *bracteatus*. Overall, our data will facilitate research on functional studies and serves as reference for other *Ananas* family members.

## Materials and Methods

### Plant materials and RNA extraction

*A*. *comosus* var. *bracteatus* was obtained from Sichuan Agricultural University garden, Sichuan Province. The plant is 2-year old which is generated from the sucker of the mother plant. The mother plants were obtained from the garden in Zhanjiang, Guangdong Province (coordinates 21°12′N 110°24′E), China [sampling place did not involve endangered or protected species and require specific permission for these locations]. Leaves with white margin and red color, roots, stem were dissected and total RNA extracted using the RNeasy plus Micro Kit (Qiagen, Hilden, Germany). The quality and quantity of total RNA was assessed at an absorbance ratio (OD_260/280_and OD_260/230_) and 1% agarose gel electrophoresis. Equal amounts of RNA from each sample were mixed into a single large pool for cDNA synthesis.

### cDNA library construction and sequencing

To obtain cDNA library, first stand synthesis cDNA was synthesized using random hexamer primers from purified Poly (A) mRNA. Second strand cDNA was synthesized using DNA polymerase I and RNase H. Short fragments were then separated by agarose gel electrophoresis and selected for PCR amplification as sequencing templates. Finally, cDNA library was sequenced on Illumina HiSeq 2000 sequencing platform, Beijing Biomarker Technologies, Beijing, China.

### Sequence analysis and *de novo* assembly

The raw reads were cleaned by removing adapter sequences, low quality sequences (reads with ambiguous bases ‘N’), and reads with >10% Q-value<20 bases. All sequences smaller than 60 bases were eliminated based on the assumption that small reads might represent sequencing artifacts [31]. The high quality reads were assembled into contigs using the Trinity method (http://trinityrnaseq.sourceforge.net/), which recovers more full-length transcripts across a broad range of expression levels and sequencing depths [32]. Subsequently, the contigs were linked into transcripts according to the paired-end information of the sequences. Based on nucleotide sequence identity, longest transcripts were considered as unigenes for functional annotation. Sequences with high nucleotide identity were screened and listed.

### Functional annotation

The assembled sequences were compared against the NCBI Nr, Nt and Swiss-port databases with an E-value < 10^-5^ [33]. The open reading frames (ORFs) were identified as the nucleotide sequence or as the protein translation using “GetORF” program of EMBOSS software package [34].We quantified transcript levels in reads per kilobase of exon model per million mapped reads (RPKM) [35]. The RPKM method is able to reflect the molar concentration of a transcript by normalizing for RNA length for the total read number in the measurement.

To annotate the assembled sequences with GO terms, the Swiss-Port BLAST results were imported into BLAST2GO, a software package that retrieves GO terms, allowing gene functions to be determined and compared [36]. The unigenes sequences were also aligned with COG database to predict and classify functions. Then KEGG (Kyoto Encyclopedia of Genes and Genomes) pathways were assigned to assembled sequences using the online KEGG Automatic Annotation Server (KAAS), http://www.genome.jp/kegg/kaas/. All the mapped sequences were annotated to the KEGG database to obtain the enzyme commission (EC) number. The EC were then mapped to the KEGG pathway to obtain the KEGG Pathway-Maps.

## Supporting Information

S1 Table Sequences with significant BLASTn matches against Nr, Swiss-Prot database and other databases. (XLSX)

S2 Table KEGG biochemical mappings for *A*. *comosus* var. *bracteatus*. (XLSX)

S3 Table Distribution of conversation between *A*. *comosus* var. *bracteatus* unigenes and pineapple EST sequences based on tBLASTX scores. (XLSX)

S4 Table Unigenes of *A*. *comosus* var. *bracteatus* with hits to rice, maize, sorghum and *Arabidopsis* based on BLASTx method. (XLSX)

S5 Table Unigenes of *A*. *comosus* var. *bracteatus* encoding key enzymes in the terpenoid and phenylpropanoid biosynthesis pathways. (XLSX)

## References

[1] Coppens d’Eeckenbrugge G, Leal F. Morphology, anatomy and taxonomy In: Bartholomew DP, Paull RE, Rohrbach KG (eds) The pineapple: botany, production and uses. CABI Publishing, Oxon, UK; 2003 pp. 13–32.

[2] Collins JL. The Pineapple, Botany, Utilisation, Cultivation. Leonard Hill Ltd, London, UK; 1960.

[3] Montinola LR. Pina. Amon Foundation, Manila, Philippines; 1991.

[4] Taussig SJ, Batkin S. Bromelain, the enzyme complex of pineapple (*Ananas comosus*) and its clinical application: an update. J Ethnopharmacol. 1998; 22: 191–203. 10.1016/0378-8741(88)90127-4 3287010

[5] Wen L, Wrolstad RE, Hsu VL. Characterization of sinapyl derivatives in pineapple (*Ananas comosus* [L] Merr.) juice. J Agric Food Chem. 1999; 47: 850–853. 10552379 10.1021/jf9808067

[6] Takata RH, Scheuer PJ. Isolation of glyceryl esters of caffeic and p-coumaric acids from pineapple stems. Lloyd. 1976; 39: 409–411.

[7] Rocha FD, Kaplan MAC. Secondary metabolites from *Ananas bracteatus* Lindley (*Bromeliaceae*). An. Acad. Bras. Ciênc. 2000; 72: 295–295.

[8] Croteau R, Kutchan TM, Lewis NG. Natural products (secondary metabolites In Buchannan B.B., Gruissem W. and Jones R.L. (eds), Biochemistry and Molecular Biology of Plant. American Society of Plant Physiologists, Rockville, MD; 2000 pp. 1250–1318.

[9] Dewick PM. Medicinal Natural Products: A Biosynthetic Approach, 2nd edn., John Wiley and Sons, Chichester; 2002.

[10] Kalaiselvi M, Gomathi D, Uma C. Occurrence of bioactive compounds in *Ananas comosus* (L.): A quality standardization by HPTLC. Asian Pac J Trop Biomed. 2012; 2: S1341–S1346.

[11] Neuteboom LW, Kunimitsu WY, Webb D, Christopher DA. Characterization and tissue-regulated expression of genes involved in pineapple (*Ananas comosus* L.) root development. Plant Sci. 2002; 163: 1021–1035.

[12] Moyle R, Crowe M, Ripi-Koia J, Fairbairn D, Botella J. Pineapple DB: an online pineapple bioinformatics resource. BMC Plant Biol. 2005a; 5: 21 16202174 10.1186/1471-2229-5-21 PMC1260026

[13] Moyle R, Fairbairn DJ, Ripi J, Crowe M, Botella JR. Developing pineapple fruit has a small transcriptome dominated by metallothionein. J Exp Bot. 2005b; 56: 101–112. 15520025 10.1093/jxb/eri015

[14] Kai T, Williams D, Spradling AC. The expression profile of purified Drosophila germline stem cells. Dev Biol. 2005; 283: 486–502. 15927177 10.1016/j.ydbio.2005.04.018

[15] Chan ET, Quon GT, Chua G, Babak T, Trochesset M, Zirngibl RA, et al Conservation of core gene expression in vertebrate tissues. J Biol. 2009; 8: 33 10.1186/jbiol130 19371447 PMC2689434

[16] Koia JH, Moyle RL, Botella JR. Microarray analysis of gene expression profiles in ripening pineapple fruits. BMC Plant Biol. 2012; 12: 240 10.1186/1471-2229-12-240 23245313 PMC3568034

[17] Ong WD, Voo LY, Kumar VS. *De novo* assembly, characterization and functional annotation of pineapple fruit transcriptome through massively parallel sequencing. PLoS One. 2012; 7: e46937 10.1371/journal.pone.0046937 23091603 PMC3473051

[18] Dohm JC, Lottaz C, Borodina T, Himmelbauer H. Substantial biases in ultra-short read data sets from high-throughput DNA sequencing. Nucleic Acids Res. 2008; 36: e105 10.1093/nar/gkn425 18660515 PMC2532726

[19] Hillier LW, Marth GT, Quinlan AR, Dooling D, Fewell G, Barnett D, et al Whole-genome sequencing and variant discovery in C. elegans. Nat Methods. 2008; 183–188. 10.1038/nmeth.1179 18204455

[20] Garg R, Patel RK, Tyagi AK, Jain M. De novo assembly of chickpea transcriptome using short reads for gene discovery and marker identification. DNA Res. 2011; 18: 53–63. 10.1093/dnares/dsq028 21217129 PMC3041503

[21] Bancroft I, Morgan C, Fraser F, Higgins J, Wells R, Clissold L, et al Dissecting the genome of the polyploidy crop oilseed rape by transcriptome sequencing. Nat Biotech. 2011; 29: 762–766. 10.1038/nbt.1926 21804563

[22] Li R, Zhu H, Ruan J, Qian W, Fang X, Shi Z et al De novo assembly of human genomes with massively parallel short read sequencing. Genome Res. 2010; 20: 265–272. 10.1101/gr.097261.109 20019144 PMC2813482

[23] Wang G, Tang W, Bidigare RR. Terpenoids as therapeutic drugs and pharmaceutical agents In Natural Products: Drug Discovery and Therapeutic Medicine. Zhang L, Demain AL, Editors. Humana Press: Totowa, NJ, USA; 2005 pp. 197–227.

[24] Fraser CM, Chapple C. The phenylpropanoid pathway in *Arabidopsis* . *Arabidopsis* Book. 2011; 9:e0152 10.1199/tab.0152 22303276 PMC3268504

[25] Vogt T. Phenylpropanoid biosynthesis. Mol Plant. 2010; 3: 2–20. 10.1093/mp/ssp106 20035037

[26] Hematy K, Cherk C, Somerville S. Host–pathogen warfare at the plant cell wall. Curr Opin Plant Biol. 2009; 12: 406–413. 10.1016/j.pbi.2009.06.007 19616468

[27] Ito Y, Shimazawa M, Akao Y, Nakajima Y, Seki N, Nozawa Y, et al Lig-8, a bioactive lignophenol derivative from bamboo lignin, protects against neuronal damage in vitro and in vivo. J Pharmacol Sci. 2006; 102: 196–204. 17031070 10.1254/jphs.fp0060711

[28] Cazarolli LH, Zanatta L, Alberton EH, Figueiredo MS, Folador P, Damazio RG, et al "Flavonoids: Prospective Drug Candidates". Mini Rev Med Chem. 2008; 8: 1429–1440. 18991758 10.2174/138955708786369564

[29] Mol J, Grotewold E, Koes R. How genes paint flowers and seeds. Trends Plant Sci. 1998; 3: 212–217.

[30] Ferreyra MLF, Rius SP, Casati P. Flavonoids: biosynthesis, biological functions, and biotechnological applications. Front Plant Sci. 2012; 3: 1e15. 23060891 10.3389/fpls.2012.00222 PMC3460232

[31] Meyer E, Aglyamova GV, Wang S, Buchanan-Carter J, Abrego D, Colbourne JK, et al Sequencing and de novo analysis of a coral larval transcriptome using 454 GSFlx. BMC Genomics. 2009; 10: 219 10.1186/1471-2164-10-219 19435504 PMC2689275

[32] Grabherr MG, Haas BJ, Yassour M, Levin JZ, Thompson DA, Amit I, et al Full length transcriptome assembly from RNA-Seq data without a reference genome. Nat Biotech. 2011; 29: 644–652. 10.1038/nbt.1883 21572440 PMC3571712

[33] Liu M, Qiao G, Jiang J, Yang H, Xie L, Xie J, et al Transcriptome Sequencing and De Novo Analysis for Ma Bamboo (*Dendrocalamus latiflorus* Munro) Using the Illumina Platform. PLoS One. 2012; 7:e46766 10.1371/journal.pone.0046766 23056442 PMC3463524

[34] Rice P, Longden I, Bleasby A. EMBOSS: the European molecular biologyopen software suite. Trends Genet. 2000; 16: 276–277. 10827456 10.1016/s0168-9525(00)02024-2

[35] Mortazavi A, Williams BA, McCue K, Schaeffer L, Wold B. Mapping and quantifying mammalian transcriptomes by RNA-Seq. Nat Methods. 2008; 5: 621–628. 10.1038/nmeth.1226 18516045 PMC13303166

[36] Conesa A, Götz S, García-Gómez JM, Terol J, Talón M, Robles M. Blast2GO: a universal tool for annotation, visualization and analysis in functional genomics research. Bioinformatics. 2005; 21: 3674–3676. 16081474 10.1093/bioinformatics/bti610

